# Mixed Manufacturer Combination With a Cementless Hemispherical Dual Mobility Cup and Polished Taper-Slip Cemented Femoral Stem: Short- to Medium-Term Results in Primary Total Hip Arthroplasty in Elderly Patients

**DOI:** 10.1016/j.artd.2025.101710

**Published:** 2025-06-12

**Authors:** Blaise Pellegrini, Alexander Antoniadis, Diane Wernly, Maya Kaegi, Julien Wegrzyn

**Affiliations:** Department of Orthopedic Surgery, Lausanne University Hospital—Centre Hospitalier Universitaire Vaudois— CHUV, Hopital Orthopedique, Lausanne, Switzerland

**Keywords:** Total hip arthroplasty, Mixed manufacturer, Elderly

## Abstract

**Background:**

There is a lack of conclusive literature regarding the mixed-manufacturer combination of the Symbol cementless hemispherical dual mobility cup (Dedienne Santé) and the Exeter V40 cemented femoral stems (Stryker) in patients who underwent primary total hip arthroplasty (THA). This study aimed to evaluate the clinical and radiographic outcomes of this combination, with a particular attention to the occurrence of dislocation and periprosthetic femoral fractures (PFFs).

**Methods:**

Between 2021 and 2023, a consecutive series of 123 primary THAs were reviewed at the latest follow-up. The mean age at surgery was 75 ± 9 ys. Postoperative complications were recorded. The clinical outcome was assessed with the Harris Hip Score. Acetabular, femoral, and global hip offset were evaluated on standard radiographs.

**Results:**

At a mean follow-up of 23 ± 7 months, the mean preoperative to postoperative Harris Hip Score improved significantly from 46 to 93 (*P* < .0001.) No dislocation was reported. No Vancouver A or B PFF was observed. One Vancouver C PFF was observed. The revision-free survival rate at 24-month follow-up was 98.6%. The global offset of the hip was restored in all the patients with a mean average increase of 3 ± 5.8 mm.

**Conclusions:**

The mixed-manufacturer combination of the Symbol cementless hemispherical dual mobility cup and the Exeter cemented femoral stem resulted in excellent short- to medium-term outcomes in patients who underwent primary THA. This combination was effective in preventing both instability and femoral Vancouver B PFF in these patients at risk, while allowing global hip offset restoration.

## Introduction

In elderly (>75 ys old) patients undergoing total hip arthroplasty (THA), main complications are: periprosthetic femoral fracture (PFF) and instability [1]. Instability still represents a major cause of THA revision with an incidence averaging 2.1% at 6 ys of follow-up and a risk of dislocation higher in patients older than 75 ys [2,3]. In addition, the quality of bone decreases with age, leading to potential intraoperative and postoperative PFF or nonoptimal stability of cementless femoral components. PFF risk was shown to be 1.9 times higher in patients older than 65 ys [4]. A diminution of the PFF risk was shown with the use of cemented stems [[5], [6], [7]]. Therefore, according to literature, hybrid THA with cemented femoral stem could be recommended as one of the best options in the elderly patients, particularly those with Dorr C type femur [[8], [9], [10], [11]]. However, no cementless true hemispherical monobloc dual mobility cup (DMC) to be used in THA construct with the cemented polished taper slip (PTS) Exeter stem is available in the portfolio of the manufacturer (Stryker, Mahwah, NJ). Consequently, a mixed-manufacturer combination of implants with the Symbol DMC (Symbol, Dedienne Santé, Mauguio, France) and the Exeter stem is routinely used in our institution for this patient population. To date, there are no published studies evaluating the outcomes of this implant combination in primary THA.

The primary objective of this study was to evaluate the clinical outcomes of a specific mixed-manufacturer implant combination, with the primary endpoint being the incidence of dislocation and PFF. We hypothesized that this implant combination would yield satisfactory outcomes in elderly patients undergoing THA for primary osteoarthritis, effectively reducing the risks of instability and PFF.

## Material and methods

Between January 2021 and January 2023, 942 THAs for primary hip osteoarthritis were performed in our institution and prospectively included in our total joint registry. In our routine practice, a cementless hemispherical DMC is systematically used in patients aged >70 ys or with ≥2 risk factors for instability [12]. In addition, in patients aged >85 ys or with Dorr C type femur, a single design of polished tapered cemented femoral stem (Exeter V40, Stryker) is systematically used to improve stability of the stem that does not rely on bone osseointegration and prevent PFF related to altered bone quality. This decision-making algorithm, introduced in 2021, identifies patients who are suitable candidates for the placement of this specific construct. Prior to its implementation, no standardized algorithm was available, and implant combinations varied for similar patients based on the preferences of senior surgeons. This algorithm was introduced to define a dedicated cemented stem with excellent long-term outcomes, which would also allow for nonhomothetic restoration of femoral offset in combination with a press-fit monoblock DMC.

In total, a consecutive series of 125 primary THAs were performed with this specific mixed-manufacturer implants combination for primary hip osteoarthritis in 113 patients (79 women [70%], mean age at surgery = 75 ± 9 ys, mean BMI = 26.8 ± 6.2 kg/m^2^, 54 patients [48%] with American Society of Anesthesiologists score ≥3). All patients met the required criteria for the use of this specific implant combination, indicating they would benefit from femoral cemented fixation and use of DMC: low bone quality as well as an age over 70 ys and/or ≥2 additional risk factors for instability. No patient was lost to follow-up. However, 2 patients passed away 5 and 9 months after THA for causes unrelated to surgery and were excluded from the study. Therefore, 123 THAs were retrospectively analyzed at a mean follow-up of 23 months. The patient’s informed consent and institutional review board approval were obtained before initiating this study (CER-VD # 2023-00,979).

All the procedures were performed through a posterolateral approach by or under the direct supervision of a senior fellowship-trained hip arthroplasty surgeon. Sizing and positioning of the THA implants were planed preoperatively on a standard calibrated anteroposterior view of the pelvis using the Traumacad software (BrainLAB AG, Munich, Germany). After hip exposure and femoral neck osteotomy, the acetabulum was exposed and reamed up to the preoperatively planed size of the Symbol cup to be used. Then, the cementless Symbol cup was implanted line to line, with the last reamer size using a press-fit technique. Positioning of the cup was in relation to the neighboring anatomic landmarks, seeking anteversion of 15° to 20° and cup abduction of 40° to 45°. The cementless Symbol DMC consists of a true hemispherical monobloc Co-Cr metal shell that is double coated with porous titanium alloy plasma spray and hydroxyapatite at the osseointegration surface without additional screw fixation. The proximal femur was then broached up to the preoperatively planed size and offset of the Exeter stem to be used, seeking anteversion of 10° to 15°. The hip was rearticulated on trial mobile bearing components, and neck length was chosen to achieve the optimal stability and leg length restoration. The internal diameter of the femoral diaphysis distal to the stem was subsequently measured, and a corresponding Exeter obturator plug was placed. The femur was then cleaned using a pulsatile irrigation (Pulsavac, Zimmer Biomet, Warsaw) and dried. The Exeter stem was then cemented in place with Exeter centralizer using 2 doses of Palacos R + G with gentamycin cement (Heraeus, Germany). Anteversion of 10° to 15° was verified, as well as the stem insertion by measuring the distance from the lesser trochanter to the end of the neck and from the prosthesis shoulder to the top of the greater trochanter. Visually, the round depth markings on the stem had to be positioned exactly as they were on the trial stem. Then, according to the age of the patient, either LFIT V40 cobalt chromium alloy (Stryker) or Biolox Delta ceramic (Stryker) femoral head was assembled with a corresponding Symbol DMC polyethylene insert (Dedienne Santé) and positioned on the stem neck. The hip was then rearticulated, and the final stability of the hip was assessed. Wound was closed, and radiographs were done postoperatively. The patients were mobilized with full weight-bearing from the day of the operation.

Preoperative outcomes were routinely assessed in our institution, including items of the Harris Hip Score [13]. Patients returned for postoperative follow-up visits at 6 wks, 3 months, 6 months, 1 year, and annually thereafter. During these visits, they underwent a clinical examination including items of the Harris Hip Score and plain anteroposterior, and lateral radiographs of the pelvis and the operated hip were taken.

Standard calibrated anteroposterior pelvic radiographs obtained before and after surgery were reviewed. The acetabular, femoral, and total offsets were computed based on preoperative and postoperative radiographs. Femoral offset was defined by the distance from the center of the femoral head to the line bisecting the long axis of the femur. Acetabular true floor offset was defined as the distance between the center of the femoral head and the true floor of the acetabulum. Global offset was calculated as femoral offset and acetabular true floor offset [14].

The radiographic limb length difference was calculated from postoperative radiographs. The teardrop was used as a landmark because it is not influenced in the vertical axis by pelvic rotation, unlike the lower aspect of the ischial tuberosities. The difference between the distances from the teardrop line to the lesser trochanters of each femur was defined as the leg-length discrepancy [15].

All demographic data, radiographic findings (calculated offsets, limb length), and Harris Hip Score (preoperative and postoperative assessments) were analyzed by descriptive statistics, including mean and standard deviation. Wilcoxon signed rank test for nonparametric paired data were used for comparison of preoperative and postoperative scores.

Statistical analyses were performed using the Xlstat software (Addinsoft, New York, NY) with a level of significance set at *P* < .05.

## Results

Two perioperative complications were reported: one weakening of the greater trochanter necessitating cerclage and one fracture of the greater trochanter which was fixed with a hook plate.

The most used stem size was size 1 (46%), followed by size 0 (28%) and 2 (19%). Only 5 size-3 and 4 size-4 stems were used. The most used stem length was the standard length of 125 mm, which was implanted in 76 THAs (62%). Implantation of a 150-mm-long stem was required in 47 cases. The 44-mm offset option was the most used (59%), with the following repartition: 37.5 mm 30%, 50 mm 9%, 56 mm 2%. A head size of 28 was implanted in 113 cases (92%), and a head size of 22 in 10 cases (8%).

No dislocation was reported. In addition, no postoperative Vancouver A or B PFF was observed. One patient (0.8%) suffered an interprosthetic Vancouver C fracture 8 months after the operation. There was no loosening of the stem associated. He underwent open reduction and internal fixation with a plate, and the evolution was favorable with an HHS score of 86 at the last clinical evaluation. One patient (0.8%) underwent revision surgery for chronic *S. epidermidis* infection. No aseptic loosening of the cup was reported. The revision-free survival rate at 24 months of follow-up was 98.6% (n = 73).

At a mean follow-up of 23 ± 7 months, the mean preoperative to postoperative HHS improved significantly from 46 ± 16 to 93 ± 9 (*P* < .0001). The subgroup of patients aged over 80 ys (n = 32) was also analyzed. A significant improvement (*P* = .0001) in the mean HHS was found, from 43 ± 16 to 89 ± 13.

The global offset of the hip was restored in all the patients with a mean average increase of 3 ± 5.8 mm (mean preoperative global offset of 78 ± 8.8 mm, mean postoperative global offset of 81 ± 8.6 mm). Preoperative acetabular offset was reduced by mean 3 ± 4.8 mm, and preoperative femoral offset was increased by mean 5.7 ± 5.9 mm.

The leg length was restored with a minor mean discrepancy of 1.7 ± 3.2 mm shorter for the operated limb. The maximal limb length discrepancy was 12 mm in favor of the nonoperated limb. Six patients exhibited a length difference exceeding 10 mm, which was already present preoperatively and was not targeted for correction in the preoperative planning. For these patients, the length difference was assessed by comparing the planned length with the postoperative outcome. No complaints about leg length discrepancy were reported.

## Discussion

The main complications of THA in elderly patients are PFF and instability. Therefore, the choice of implant combination should address these issues in the most effective way. To our knowledge, no study evaluated the outcome of the off-label combination of the Symbol monobloc cementless DMC (Dedienne Santé) and cemented polished tapered Exeter stem (Stryker). Therefore, this study aimed to evaluate the outcome of the mixed-manufacturer implants combination at a mean 2-year follow-up.

Regarding instability, DMC have been proved to prevent dislocation after THA compared to conventional single mobility acetabular components, especially in patients with high dislocation risk [16]. A recent review by Martino et al. [17] found a mean rate of dislocation for primary DMC THA of 0.9%. Tigani et al. [18] analyzed the Registry of Prosthetic Orthopedic Implants of Emilia Romagna, a regional Italian registry of 129,910 primary THAs and found that DMC THA had a statistically significant lower risk of revision for dislocation (hazard ratio = 1.6) than single-cup THA with a head diameter of 22-28 mm. DMC exist in monobloc or modular form. In our opinion, the use of a monobloc DMC might be the preferred option over a modular one. Modular DMC are more expensive with potential for fretting corrosion [19] and induce a reduced jumping distance with decrease of the inner diameter of the cup [20]. A study of Gkiatas et al. [21] showed that elevated levels of serum cobalt ions were present in 5.2% of patients at a mean follow-up of 27.4 months in patients with modular DMC. In parallel, they showed in another study an overall survivorship of the acetabular component of 99% at minimum 5 ys of follow-up in a cohort of 200 monobloc DMC THAs [22]. The advantage of modular DMC is to allow screw fixation in case of an acetabular compromised bone stock with inadequate press-fit fixation. However, in this situation, direct cementation of monobloc DMC might be considered with excellent results reported in the literature [23]. Hemispherical Symbol DMC already proved effectiveness to prevent instability in a multicentric consecutive cohort of 332 patients undergoing primary THA [24]. The main finding of our study was that no dislocation was reported in our cohort, although most patients (60%) were presenting ≥ 2 risk factors for dislocation.

Regarding dislocation, it is well established that restoring or increasing the femoral offset by up to 5 mm enhances the stability of the prosthetic hip [14,25]. A stem offering multiple options for offset restoration should therefore be selected in combination with the use of monobloc DMC. In our study, the mean preoperative to postoperative change in femoral offset was reported at 5.7 mm with the use of the Exeter stem.

Regarding PFF, the use of a cemented stem is associated with the reduction of PFF risk [6,7]. Analysis of the American Joint replacement registry found cementless stem fixation in patients aged over 65 ys was associated with increased risk of all-time PFF (hazard ratio of 7.7) [5]. Mitigating this risk is of paramount importance as patients within this age group undergoing revision surgery in the first year exhibit the highest mortality risk [26]. Some studies suggest that collared cementless stems could reduce the risk of PFF when comparing various models of uncemented femoral stems [[27], [28], [29], [30]]. Nevertheless, there is still a lack of long-term studies on overall survival with these collared stems to confirm that they offer better overall survival in elderly patients. Literature shows that cemented femoral fixation in patients older than 75 ys provides the lowest risk of revision [26,31,32]. There are 2 main designs for cemented stems, each with its own advantages: PTS vs composite beam stem. Taper slip stems generate forces on the femoral bone that may increase the risk of fracture; however, this also protects the stem against loosening and osteolysis. Thien et al. [33] analyzed the Nordic arthroplasty register association database and found that stem survival at 14 ys was only marginally different for the polished tapered Exeter stem compared to the composite beam Lubinus stem (94.5% ± 1% vs 95.4% ± 0.6%) despite a higher initial rate of PFF for the PTS. The Exeter stem remains the most used cemented femoral stem according to joint registries over the last 50 ys. Studies show low stem revision rates ranging from 1.9% to 3.8%, with extremely low rates of aseptic loosening (0.22%) [[34], [35], [36]]. No Vancouver type B fractures were reported in our cohort, although a significant portion of the stems used are size 0 stems, which have been associated with a higher risk of PFF than other sizes [37]. In our study, only one PFF occurred (0.8%). It was an interprosthetic/Vancouver type C periprosthetic diaphyseal spiroid fracture in an 89-year-old patient with an ipsilateral total knee arthroplasty that was implanted in 2014. The patient underwent open reduction and internal fixation with an non-contact bridging plate and did not require a revision of the hip arthroplasty. It has been demonstrated that the presence of a knee prosthesis influences the occurrence and mechanism of an interprosthetic fracture [38], and literature suggests that the design of the stem does not influence the risk of Vancouver C fracture observed with the Exeter stem in comparison to composite beam designs such as the Lubinus stem [39]. The hypothesis was proposed that the double mobility mechanism, by restricting potential dislocation, imposes more stress on the femur and increases the rate of fractures in certain situations [40]. Recently a study by Hogget et al. [41] showed no difference in the postoperative fracture rate comparing dual mobility with conventional acetabular bearings.

The rationale for the off-label combination of the Exeter stem and the Symbol cup is that the unique V40 femoral neck of the Exeter stem that is thin, cylindrical, and highly polished was biomechanically demonstrated by Wegrzyn et al. [42] to be the most suitable femoral neck design to be used with a DMC in order to reduce damage lesions and wear of the polyethylene insert particularly at the third articulation. The off-label combination of implants issued from different manufacturers is a common practice during THA although the medicolegal implications of this practice are not yet fully established [43,44]. In Europe to date, no orthopedic surgeon has been held legally responsible or ended up in a lawsuit for the use of mixed components, according to a review of Peters et al. [45]. However, Peters et al. state that "the unauthorized mixing of components can create a liability risk based on European and national law. An orthopedic surgeon who mixes components from different manufactures could qualify as a 'manufacturer of a finished product' and may be held liable without fault if the product appears to be defective." An analysis of data from the National Joint Registry of England and Wales demonstrated that the most favorable outcome achieved by the Exeter stem was with the off-label combination with the Charnley Elite cup [46]. Holland et al. [47] recently demonstrated that dual mobility THA with mixed manufacturers was a viable option, based on a series of 103 mixed-manufacturer DM THA. Wakeling et al. [48] published the only series concerning the mixed-manufacturer Symbol–Exeter association with a DMC and found a dislocation rate of 6.9%, including one case of intraprosthetic dislocation. The series, however, presents 2 main differences from ours, making comparison challenging: Most of their cases were revisions (58%), and the cup used was a SERF Novae DMC (SERF, Decines, France). Furthermore, among the primary THAs, 8 had been performed for fractures, either of the femoral neck or acetabulum. Thus, their series does not allow for interpretation of outcomes regarding primary THAs performed in an elective context. Our series has a mean follow-up of 23 months and shows a revision-free survival rate at 24 months follow-up of 98.6%; however, this already demonstrates a low complication rate as the 2023 Swiss Implant Registry rapports a 2-year revision rate of 2.5% for primary osteoarthritis THA, with the vast majority of revisions occurring within the first 3 months postoperatively, notably for PFF and instability [49]. Regarding the long-term survival rate of monoblock DMC implants, a recently published study demonstrated an excellent survival rate of 98.6% at a minimum follow-up of 10 ys [50] in primary THA. The combination of monoblock DMC with a stem such as the Exeter, whose long-term durability is well established, appears to offer promising long-term outcomes.

The main limitation of our study is the relatively short follow-up duration. Even though most complications occur within the first 3 months, a more extended follow-up is essential to gain insights into long-term outcomes. This is particularly crucial for estimating rates of certain complications that may manifest at a later stage, such as aseptic loosening or intraprosthetic dislocation. Indeed, the latter one typically occurs on average 9 ys after prosthesis implantation and is predominantly due to progressive phenomena such as arthrofibrosis, heterotopic ossification, and cup loosening [51]. Aseptic loosening is one of the few complications to exhibit a linear incidence and therefore occurs at a greater distance from implantation as demonstrated by the 2023 analysis of the Swiss National Registry of Implants [49].

## Conclusions

The mixed-manufacturer implant combination of the Symbol cementless hemispherical DMC and the Exeter cemented femoral stem resulted in excellent short- to medium-term outcomes in elderly patients who underwent THA for primary hip osteoarthritis. This combination was effective to prevent both instability and femoral Vancouver B PFF in these patients at risk.

## Conflicts of interest

JW receives royalties from Dedienne santé; is in the Speakers bureau/gave paid presentations for Dedienne santé, Enovis, DePuy, and Stryker; is a paid consultant for Stryker and Enovis; and is in the medical/orthopedic publications editorial/governing board of the *Journal of Arthroplasty* and *Swiss Medical Weekly*. All other authors declare no potential conflicts of interest.

For full disclosure statements refer to https://doi.org/10.1016/j.artd.2025.101710.

## CRediT authorship contribution statement

**Blaise Pellegrini:** Writing – review & editing, Writing – original draft, Visualization, Software, Resources, Project administration, Methodology, Investigation, Formal analysis, Data curation, Conceptualization. **Alexander Antoniadis:** Writing – review & editing, Visualization, Validation, Supervision, Methodology, Conceptualization. **Diane Wernly:** Writing – review & editing, Validation, Supervision, Resources, Project administration, Methodology, Investigation, Formal analysis, Data curation, Conceptualization. **Maya Kaegi:** Writing – review & editing, Visualization, Validation, Supervision, Project administration, Methodology, Investigation, Formal analysis, Conceptualization. **Julien Wegrzyn:** Writing – review & editing, Visualization, Validation, Supervision, Project administration, Methodology, Investigation, Formal analysis, Conceptualization.

## Appendix A Supplementary data

Conflict of Interest Statement for Pellegrini

Conflict of Interest Statement for Wernly

Conflict of Interest Statement for Wegrzyn

Conflict of Interest Statement for Antoniadis

Conflict of Interest Statement for Kaegi
