# Supplementary material for: Mixed Manufacturer Combination With a Cementless Hemispherical Dual Mobility Cup and Polished Taper-Slip Cemented Femoral Stem: Short- to Medium-Term Results in Primary Total Hip Arthroplasty in Elderly Patients
Source: Arthroplast Today. 2025 Jun 12;33:101710. doi: 10.1016/j.artd.2025.101710 (PMC12192544; doi:10.1016/j.artd.2025.101710)
Supplement: Conflict of Interest Statement for Wegrzyn [file mmc3.pdf]

# CONFLICT OF INTEREST STATEMENT

## *American Association of Hip and Knee Surgeons*

(Adopted from the American Academy of Orthopaedic Surgeons disclosure statement)

The following form **must be filled out completely and submitted by each author (example, 6 authors, 6 forms). All items require a response. If there is no relevant disclosure for a given item, enter "*None.*"**

Mixed manufacturer combination with the Symbol® cementless hemispherical dual mobility cup and the Exeter® V40 cemented femoral stem. Short- to medium-term results in primary total hip arthroplasty in elderly patients.

Manuscript Title

1. Royalties from a company or supplier (The following conflicts were disclosed): Dedienne santé

2. Speakers bureau/paid presentations for a company or supplier (The following conflicts were disclosed): Dedienne santé, Enovis, DePuy, Stryker

3A. Paid employee for a company or supplier (The following conflicts were disclosed): NONE

3B. Paid consultant for a company or supplier (The following conflicts were disclosed): Stryker, Enovis

3C. Unpaid consultants for a company or supplier (The following conflicts were disclosed): NONE

4. Stock or stock options in a company or supplier (The following conflicts were disclosed): NONE

5. Research support from a company or supplier as a Principal Investigator (The following conflicts were disclosed): NONE

6. Other financial or material support from a company or supplier (The following conflicts were disclosed): NONE

7. Royalties, financial or material support from publishers (The following conflicts were disclosed): NONE

8. Medical/Orthopaedic publications editorial/governing board (The following conflicts were disclosed): Journal of Arthroplasy, Swiss Medical Weekly

9. Board member/committee appointments for a society (The following conflicts were disclosed): NONE

**Each author must sign AND print or type his/her name, date and submit a separate form**

In addition, one BLINDED Conflict of Interest form (no author names used) should be submitted per manuscript with all author disclosures.

| Wegrzyn | Julien Wegrzyn | 11.24.2024 |
|---|---|---|
| Author Name (Print or Type) | Author Signature | Date |
